# Can Genetic Estimators Provide Robust Estimates of the Effective Number of Breeders in Small Populations?

**DOI:** 10.1371/journal.pone.0048464

**Published:** 2012-11-06

**Authors:** Marion Hoehn, Bernd Gruber, Stephen D. Sarre, Rebecca Lange, Klaus Henle

**Affiliations:** 1 UFZ - Helmholtz Centre for Environmental Research, Department of Conservation Biology, Leipzig, Germany; 2 Institute for Applied Ecology, University of Canberra, Bruce, Australian Capital Territory, Australia; Biodiversity Insitute of Ontario - University of Guelph, Canada

## Abstract

The effective population size (*N_e_*) is proportional to the loss of genetic diversity and the rate of inbreeding, and its accurate estimation is crucial for the monitoring of small populations. Here, we integrate temporal studies of the gecko *Oedura reticulata,* to compare genetic and demographic estimators of N_e_. Because geckos have overlapping generations, our goal was to demographically estimate *N_bI_*, the inbreeding effective number of breeders and to calculate the *N_bI_/N_a_* ratio (*N_a_* = number of adults) for four populations. Demographically estimated *N_bI_* ranged from 1 to 65 individuals. The mean reduction in the effective number of breeders relative to census size (*N_bI_/N_a_*) was 0.1 to 1.1. We identified the variance in reproductive success as the most important variable contributing to reduction of this ratio. We used four methods to estimate the genetic based inbreeding effective number of breeders *N_bI(gen)_* and the variance effective populations size *N_eV(gen)_* estimates from the genotype data. Two of these methods - a temporal moment-based (*MBT*) and a likelihood-based approach (*TM3*) require at least two samples in time, while the other two were single-sample estimators - the linkage disequilibrium method with bias correction *LDNe* and the program *ONeSAMP*. The genetic based estimates were fairly similar across methods and also similar to the demographic estimates excluding those estimates, in which upper confidence interval boundaries were uninformative. For example, *LDNe* and *ONeSAMP* estimates ranged from 14–55 and 24–48 individuals, respectively. However, temporal methods suffered from a large variation in confidence intervals and concerns about the prior information. We conclude that the single-sample estimators are an acceptable short-cut to estimate *N_bI_* for species such as geckos and will be of great importance for the monitoring of species in fragmented landscapes.

## Introduction

The effective population size (*N_e_*), which is inversely related to the rate of loss of genetic diversity through genetic drift and inbreeding [1], is one of the most essential parameter in population genetics, evolutionary ecology, and conservation biology [2], [3], [4], [5], [6], [7]. Habitat fragmentation leads to small, isolated populations that are susceptible to the fixation of deleterious alleles, inbreeding depression, and local extinction [8]. In addition, *N_e_* is affected by life histories, population dynamics, and demographic history of individuals [9], [10], [11], [12] and the exact nature of the relationship between demographic and genetic *N_e_* is unknown and will vary between species and populations. Monitoring *N_e_* would provide an excellent tool for monitoring the status of fragmented populations of conservation concern [7], [11], [12].

Effective population sizes have been estimated using both genetic and demographic approaches, but only rarely in combination [10], [13], [14]. Primarily, this is because it has been extremely difficult to estimate demographic effective population size *N_e(demo)_* in natural populations. However, recent developments in theory and in analytical tools have simplified the analysis of demographic parameters and made possible more robust estimation of *N_e(demo)_* [2], [3], [15], [16], [17], [18], [19].

In populations with overlapping generations, demographic based approaches usually focus on estimation of the effective number of breeders, which is the effective size of a breeding population in a given year [2]. Estimating *N_e_* can be complex because methods that account for age structure either require long-term studies in which a population is sampled annually for several generations [20] or restrictive assumptions (such as stable age structure, age-independent variance in reproductive success, random mortality within all age classes) must be met when analysing the data [18], [19], [21], [22].

Variation in four main life history and population traits (sex ratio; variance in individual reproductive success; age structure; and fluctuating population size) can reduce the effective number of reproducing individuals *N_e(demo)_* below the number of sexually mature adults (*N_a_*) in a population [2], [3], [10], [13], [18], [23]. Of these variables, estimating the variance in individual reproductive success and accounting for age-structure provide particular challenges. In the past, variance in individual reproductive success could only be obtained from intensive long-term studies of individually known animals [9]. More recently, polymorphic genetic markers, such as microsatellite loci, have been used to measure individual reproductive success by directly assigning offspring to parents through the comparison of genotypes [13], [17], [24], [25], [26]. The genetic approach can be enormously powerful, but failing to sample all potential parents and assignment errors can potentially bias the estimate of relative reproductive success [27], [28].

Numerous procedures for estimating *N_e(gen)_* from genetic data have been developed [26], [29], [30], [31], [32], [33], [34], [35], [36], [37]. By far the most common is the temporal approach, which estimates variance effective population size *N_eV_* and is based on the idea that allele frequencies will differ between samples taken at two different times from the same population as a result of random genetic drift. The amount by which they will differ is inversely proportional to effective population size. In addition to the F-statistic estimators [2], [23], [38], [39] several likelihood-based approaches have been developed, which require temporal data, but have increased accuracy because they incorporate a greater proportion of the available data [29], [35], [40]. Surprisingly, methods for estimating the effective population size that require only a single sample in time have only been applied in recent years because they were often imprecise and biased [30], [32], [33], [34]. Accurate single sample approaches would be advantageous in the analysis of long-lived species for which two or more samples collected at least a generation apart may take many years or even decades to gather [41]. The standard linkage disequilibrium method (*LDNe*) [30], [34], [42] is the most frequently used single sample method. Another recently developed point estimator is implemented in the program *ONeSAMP* and uses summary statistics and approximate Bayesian computation to estimate effective population size [33]. If a single cohort has been sampled, both methods are directly related to inbreeding *N_bI_*, which predicts the rate of decrease in heterozygosity. However, their relationship to *N_bI_* and *N_e_* is less clear should more than a single cohort of adults be sampled, as in this study [43] and neither method should be expected to exactly reproduce *N_bI_* Variance and inbreeding *N_e_* can also converge in empirical studies and are usually not distinguished in practice [4], [44]. However, they will differ depending on whether populations are increasing or decreasing [45].

Empirical tests of numerous genetic approaches to estimating *N_e_* and *N_b_* are essential if we are to fully evaluate their usefulness in different situations. Such evaluations are difficult, not least of all for species with long life spans and slow reproductive histories because obtaining the necessary temporal population demographic and genetic information as well as estimates of reproductive success is extremely difficult. Here, we report on a comparative empirical study in which we estimate *N_a_* (adult population size) in four Western Australian populations of the reticulated velvet gecko (*Oedura reticulata*). We take advantage of extensive long-term demographic data [46], [47], [48] and microsatellite DNA analyses to estimate the genetic temporal and single-sample effective population size. We also estimate the inbreeding number of breeders using the demographic parameter *N_bI_* and assess which parameters are responsible for the decrease relative to census size. We focus on estimating *N_b_* because the estimation of *N_e_* is extremely difficult for long live species [2]. Specifically, it would require that the life-time reproductive success of all individuals in each population be known. Those data are not available for the populations in question.

As a result of similar territorial behaviour in male and female geckos, we predict that sex ratio will influence the inbreeding number of breeders (*N_bI_*) only marginally and that variance in reproductive success will reduce *N_bI_* more strongly. As part of our analysis, we use an approach to estimating the variance in reproductive success that accounts for incomplete sampling. Finally, we evaluate the genetic estimates of *N_bI_* and *N_eV_* derived from single and temporal population microsatellite studies against demographic based population estimates of *N_bI_* and adult population size *N_a_*. We base this evaluation on whether the genetic estimates could be obtained and were consistent among methods and whether confidence intervals were informative.

## Methods

### Study Species

*Oedura reticulata* is endemic to the southwest of Western Australia and is a habitat specialist. It is limited in its range of habitat, being exclusively arboreal and restricted to smooth-barked *Eucalyptus* woodlands [49]. Males mature one year earlier than females, usually reproducing for the first time at an age of 3.8 years (compared with 4.8 years in females) but adult mortality is similar for both sexes [46], [49].

### Study Area and Sampling

**Figure 1:**
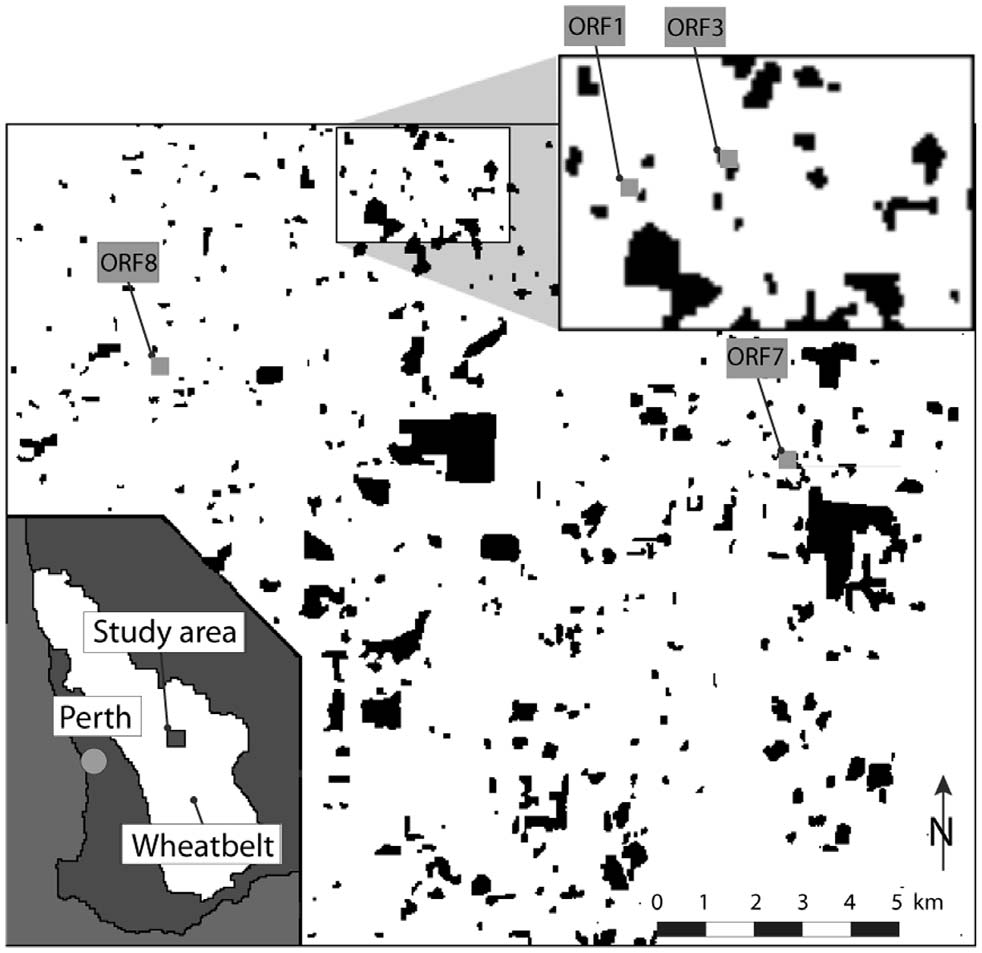
Map of the study area and location of sites in the Western Australian wheatbelt. O. reticulata populations inhabit fragments ORF1, ORF3, ORF7, and ORF8.

The study area is located between Kellerberrin and Trayning in the Western Australian wheatbelt. Large areas of native vegetation have been removed from this region and replaced by agricultural crops, pastures, and livestock. Since 1900, approximately 93% of the original vegetation has been cleared, with the remnant vegetation distributed over thousands of patches of varying size. The study species occurs in small woodland remnants that are separated from other remnants by expanses of land cleared for agriculture. The species has been subjected to severe population fragmentation [50]. Four populations (ORF1, ORF3, ORF7, ORF8) were selected (Figure 1) for comparative purposes based on the fact that tissue samples and demographic data were collected concurrently in time [51], [52], [53], [54].

The field sampling and population estimates were conducted from 1989 to 1991 and from November 2000 to March 2002. Geckos were spotted at night using head-torches and captured by hand during 5–7 sampling sessions (consecutive nights). Each animal was marked individually by clipping the tip of the toe, a standard procedure in lizards [55], [56], for which no negative effects have been detected in other studies [57], [58]. Each sampling session lasted for up to four hours after sunset. The tip of the tail of each individual sampled was removed and stored in liquid nitrogen. Snout-vent length, body mass, sex, and age were recorded for all individuals. Sex can be accurately determined based on the shape of the tail base and the presence/absence of pre-anal pores in sexually mature individuals of both species. All procedures involving animals were carried out according to the ‘Australian Code of Practise for the Care and Use of Animals for Scientific Purposes’ and were approved by the University of Canberra Animal Ethics Committee (CEAE # 02/13 and 05/17). Permits to take Fauna was given by the Department of Conservation and Land Management (CALM Licence # SF004040.).

### Census Population Size

We estimated adult (*N_a_*) and adult female (*N_f_*) population size with models for closed populations, using program *CAPTURE*. In studies with adequate data, the assumptions about capture probability are more flexible when using *CAPTURE*. We estimated adult male population size (*N_m_*) as the difference of the estimates of *N_a_* and *N_f_*. The closure assumption required by *CAPTURE* is reasonable because movement between isolated habitat fragments is rare [50], [54] and deaths are unlikely in the time span of one week given the high life expectancy of the gecko species [46]. *CAPTURE* incorporates seven models that allow for time-dependence (model M_t_), trap response (model M_b_), and individual variation (model M_h_) of capture probabilities as well as all combinations thereof, except for model M_tbh_ [59]. The discriminant function of *CAPTURE* to select an appropriate estimation model has limited performance, especially when population sizes are small [60], [61]. Therefore, we also inspected individual model tests and whether confidence intervals were suspiciously small. Individual tests and model selection suggested model M_0_ in most cases. In the six remaining cases the confidence intervals of the selected models were suspiciously small and no biological evidence was available that justified differences in the capture process. Therefore, we used M_0_ for all data sets.

### Demographic Estimates of N_bI_

The inbreeding effective number of breeders (*N_bI_*) depends on sex ratio and the number (*k*) of offspring produced by the potential parents and its interindividual variance (*σ_k_^2^*) in a particular year. The estimates were made in a stepwise fashion to assess how much these factors contribute to a reduction of *N_bI_* compared to census population size. The effects of **unequal sex ratios** can be accounted for with the equation [22]:
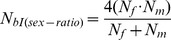
(1)with *N_f_* and *N_m_* signifying the number of adult females and males, respectively.

To account for **variance in reproductive success** among individuals, *N_f_* and *N_m_* in equation (1) need to be replaced by the inbreeding effective number of males and females (*N_bm_, N_bf_*) in a given year [10, equation 2–4]:
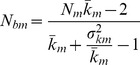
(2)and
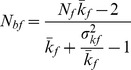
(3)to get the inbreeding number of breeders for year i:
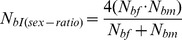
(4)


### Estimates of Mean and Interindividual Variance in Reproductive Success (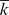 and σk2)

We estimated 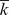 *and σ_k_^2^* using parentage and mating system analysis [17], [24], [25]. We obtained our parentage data from an independent microsatellite DNA study of mating systems in four populations of *O. reticulata* (Lange and Hoehn unpubl.). Of the four populations, two (ORF3 and ORF7) are common to the populations of the current study. Microsatellite genotypes of nine loci were determined for all candidate parents from 423 individuals captured over a three months period. We incorporated the data into program *CERVUS 3.0* [62], [63], which calculates log-likelihood ratio scores for parentage. Statistical confidence (Delta) was estimated for critical values at strict (95%) confidence levels based on computer simulation of paternity inference with allele frequencies from the population under study. Simulation parameters were as follows: 100,000 cycles, 100% of loci typed with an error rate of 0.01, and 0.8 of candidate parents sampled. Individual reproductive success was recorded as the total number of offspring (juveniles) assigned to each parent in the above analysis.

For the two populations shared with the independent mating study, we used the parentage estimates for these two populations. For the remaining two populations we used the weighted mean of the mean number of offspring of the four populations, for which parentage analyses were made, with the number of potential parents analysed as weights. For variances, we used the weighted mean of the within sample variances of the four populations with parentage data [64].

Estimates from parentage analysis have unknown bias unless all individuals in the pool of potential breeders of the next generation are sampled, genotyped, and reliably assigned to their parents. We therefore regard estimates obtained from genetic parentage analysis unadjusted estimates, 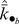 and 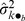; the dot indicates male or female. If sampling probability is constant and random, then unadjusted estimates and estimates adjusted for a sampling probability less than 1 (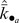 and 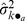) will result in the same estimate of the inbreeding effective number of breeders [65], [66]. Nevertheless, this is an approximation. When population size and the mean number of offspring are small, and/or the proportion of offspring sampled and assigned to a parent is low, estimates may differ considerably. Therefore, we corrected the estimates from the parentage analyses. In addition, weighted means across populations can only be calculated with corrected *k*, unless the percentage *p* of juveniles sampled and successfully assigned to parents would have been identical across populations. Adjusted values of the mean number of offspring can be obtained by noting that:
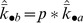
(5)


The percentage *p* of juveniles sampled and successfully assigned to parents can be calculated as:
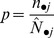
(6)with 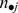 being the number of juveniles sampled and successfully assigned to a parent and 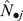 being the estimated total number of successfully assigned offspring produced by all sampled parents. The dot indicates male or female parent. Offspring that didn’t assigned to a parent was disregarded when estimating the number of offspring. We estimated 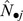 for each population with the Lincoln-Peterson method, which is preferable to methods implemented in CAPTURE, if the number of individuals is small [60].

To obtain adjusted individual variances of the number of offspring, we use the relationship between the variance at an earlier and the variance at a later stage derived by Crow and Morton (1955) [65] under the assumption of random survival (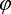). The assumption that all juveniles had the same probability of being sampled and successfully assigned to parents is equivalent to the assumption of random survival so that *p* can be substituted for 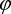 in the equation and resolve for 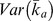.
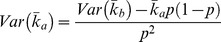
(7)


### Microsatellite DNA Analysis for Estimating N_bI(gen)_ and N_eV(gen)_

DNA was extracted from the tip of the tail of each individual using the Chelex extraction method [67]. We genotyped individuals of *O. reticulata* using nine tetranucleotide microsatellite loci (OR205, OR220, OR266, OR6F4, OR10H7, OR11G3, OR12D7, OR12D9, OR14A7) described in Hoehn and Sarre [68], [69]. Multiplexed PCR products of all nine loci were analysed by capillary electrophoresis on a CEQ8000 (Beckman-Coulter). DNA size standard 600 (Genome Lab) was included within each sample, allowing accurate sizing of alleles and comparison between analyses. Results were analysed with fragment analysis software version 8.0.52 (Beckman-Coulter). *FSTAT 2.9.3* [70], [71] and *GENEPOP 3.2a* [72] were used to calculate descriptive statistics and departure from Hardy-Weinberg equilibrium by comparing the observed inbreeding coefficient (*F_IS_*) with a distribution of 1000 bootstrap replicates using *FSTAT 2.9.3*. Additionally, evidence for the presence of null alleles at each locus within each population was assessed using the program *MICROCHECKER* v 2.2.3 [73].

### Estimation of the Effective Population Size N_bI(gen)_ and N_eV(gen)_

We used four methods of analysis to estimate *N_bI(gen)_* and *N_eV(gen)_* from the genotype data of *O. reticulata*. Two of these methods require at least two samples in time – a temporal moment-based (*MBT*) and a likelihood-based approach (*TM3*) implemented in the program *NeEstimator* [74]. We chose *MBT* as it is a standard approach, which has been used in a wide range of studies [2], [3], [10], [38], [75], [76]. The *TM3* approach was chosen since it was assumed to perform satisfactorily with data from populations with small *N_eV_* [29]. For the point-in-time approach we also used two methods. We used the linkage disequilibrium method with bias correction as implemented in *LDNe* [30], [34], [41]. The standard linkage disequilibrium method [42] was shown to be biased [32], so we apply the empirical correction developed by Waples [41]. We calculated estimates assuming random mating and excluded all frequencies lower than 0.05. We used a jackknife procedure to construct 95% confidence intervals. We also estimated *N_bI_* with program *ONeSAMP* 1.1 [33], which uses summary genetic statistics for approximate Bayesian computation. The upper and lower bounds of the prior distribution in all populations were 2 and 250, respectively. Priors of 2–100 and 10–250 were also tested to assess whether the results were robust to changes in these assumed values.

## Results

### Adult Population Size N_a_

**Figure 2:**
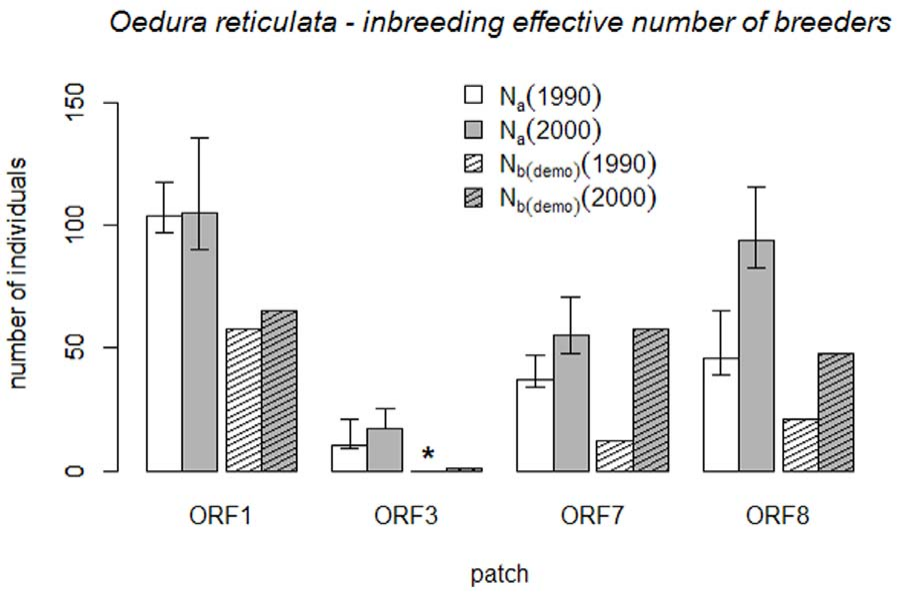
Comparison of estimates of adult population size 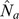 and of inbreeding effective number of breeders 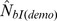. The comparison is shown between 1990 and 2000 for four populations of *O. reticulata*. Site codes follow Table 1.

We observed a significant increase in the size of two populations of *O. reticulata* over the ten years period (Table 1, Figure 2) while the remaining two populations remained more or less stable. In particular, the population in fragment ORF8 doubled in size from 46 to 94 individuals.

**Table 1:** Estimates of adult (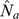)and adult female (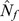) population sizes, and the inbreeding number of breeders when accounting only for sex ratio of O. reticulata (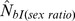).

Frag	Ref	Year	Samp	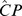	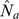	LCI	UCI	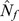	LCI	UCI	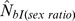	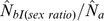
ORF1	170	1990	7	0.24	104	97	118	68	58	77	91	0.88
ORF1	170	2000	7	0.20	105	90	136	62	52	71	99	0.95
ORF3	s84	1990	6	0.24	10	9	21	7	4	10	8	0.81
ORF3	s84	2000	5	0.37	17	17	25	12	8	15	14	0.80
ORF7	168	1990	7	0.25	37	34	47	22	16	28	35	0.94
ORF7	168	2000	7	0.29	55	48	71	29	21	36	54	0.99
ORF8	175	1990	6	0.26	46	39	65	24	17	31	45	0.99
ORF8	175	2000	6	0.24	94	83	116	55	46	64	89	0.95
					59							0.91

Habitat fragments sampled and reference number (Ref) referring to the study of Sarre et al. (1995). Samp: number of sampling occasions, 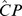: daily capture probability: LCI and UCI: lower and upper 95% confidence interval.

### Demographic Estimates of Inbreeding N_bI_

#### Influence of sex ratio on N_bI(demo)_

The sex ratios within the *O. reticulata* populations were slightly biased towards females with an average 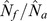 = 0.61. This bias resulted in minor reductions in the estimated inbreeding number of effective breeders 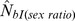 with the mean 
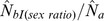
 ratio over all populations being 0.91. The reduction was highest in fragment ORF3 (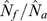 = 0.80) with a very small adult population size and biased sex ratio in both study periods.

#### Influence of variance in reproductive success on N_bI(demo)_

The number of offspring per female in the mating study ranged from 0 to 2, with a mean number 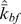 = 0.57 and variance 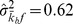 in population ORF1 and with 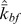 = 0.11 and variance 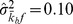 in ORF7. The number of offspring per male also ranged from 0 to 2, with 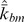 = 0.41 and variance 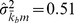 in ORF1 and 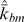 = 0.15 and variance 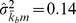 in ORF7. Corrected estimates of 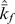 ranged from 0.11 to 0.65 with 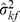
ranging from 0.10 to 0.72 in females. In males, 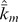 ranged from 0.15 to 0.49 and 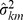 ranging from 0.14 to 0.64 (Table 2). The inbreeding effective number of females for both years ranged from 1 to 55 and for males from 0 to 31. For population ORF3, no *N_bm_* estimate (1990) was obtained due to the small number of offspring. Variance in reproductive success caused a considerably stronger reduction of the number of breeders than sex ratio, with the mean 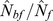 = 0.43 and 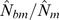 = 0.46 in 1990 and 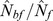 = 0.62 and 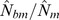 = 0.60.

**Table 2:** Estimates of reproductive parameters and the demographic effective inbreeding number of breeders (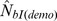) for four populations of *O. reticulata* and its comparison to census population size (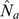).

Frag	Year	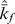	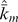	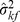	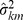	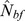	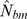	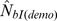	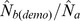
ORF1	1990	0.65	0.49	0.72	0.64	55	20	58	0.6
ORF1	2000	0.65	0.49	0.72	0.64	50	24	65	0.6
ORF3	1990	0.36*	0.45*	0.48*	0.56*	1	–**	–**	–**
ORF3	2000	0.36*	0.45*	0.48*	0.56*	3	0	1	0.1
ORF7	1990	0.11	0.15	0.10	0.14	9	5	12	0.3
ORF7	2000	0.11	0.15	0.10	0.14	27	31	58	1.1
ORF8	1990	0.36*	0.45*	0.48*	0.56*	9	12	21	0.5
ORF8	2000	0.36*	0.45*	0.48*	0.56*	25	23	48	0.5

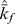
 and 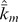
are the estimated corrected mean number of juveniles produced by female and male potential breeders and 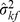 and 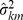
are the estimated corrected inter-individual variances (see methods). 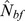 and 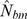 are the inbreeding effective number of females and males.*: estimates based on the weighted mean from four populations (see methods). **: no estimates possible due to few numbers of offspring.

#### Inbreeding effective number of breeders N_bI(demo)_

The inbreeding effective number of breeders ranged from 12 to 58 in 1990 and from 1 to 65 in 2000. ORF3 occupied the smallest and ORF1 the largest inbreeding effective numbers of breeders (Table 2). The overall mean influence of sex ratio and variance in reproductive success on the ratio of the inbreeding effective number of breeders relative to census population size (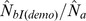) ranged from 0.1 to 0.6 with the exception of ORF7 where the ratio was 1.1.

### Genetic Analyses for the Estimation of N_bI(gen)_ and N_eV(gen)_

A total of 228 *O. reticulata* were genotyped at nine microsatellite loci. We analysed between 24 and 30 randomly selected individuals from each of the two temporal samples from ORF1, ORF7, ORF3, and ORF8. The DNA amplification had a high success rate, even for the tail samples taken in 1990. *MICROCHECKER* v 2.2.3 [73] revealed no evidence of null alleles in any of the *O. reticulata* loci.

### Genetic Estimates of N_bI(gen)_ and N_eV(gen)_

The genetic estimates of *N_bI(gen)_* and *N_eV(gen)_* gave fairly similar results among methods excluding those estimates, in which upper confidence interval boundaries were uninformative (Table 3). The moment-based temporal *MBT* method gave estimates for all populations but upper confidence intervals could be constructed for two estimates only. The likelihood-based *TM3* method also created estimates for all populations. However, one estimate did not produce upper confidence intervals below the boundary imposed as input value. In addition, the maximum *N_eV_* allowed substantially influenced the results of the estimations and confidence intervals (data not shown). The linkage disequilibrium method *LDNe* and *ONeSAMP* generated fairly consistent estimates within populations across years, except for the *LDNe* estimate in ORF8, where upper confidence intervals were not obtained in 1990. Upper and lower confidence intervals were obtained for all *ONeSAMP* estimates and were narrower than confidence intervals from other methods. The results were not sensitive to the prior.

**Table 3:** Genetic based estimates of effective population size 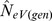 (MBT, TM3) and 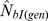
(LDNe, ONeSAMP) and comparison with the demographic estimates of the inbreeding effective number of breeders 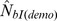 for four populations of *O. reticulata.*

Frag	Ref	Year	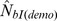	*MBT*	*TM3*		*LDNe*	*ONeSAMP*
ORF1	170	1990	58	243 (52 - **∞)**	64 (30–242)	55 (31–156)		37 (32–50)
ORF1	170	2000	65			34 (21–73)		37 (32–56)
ORF3	s84	1990	–**	45 (17- 364)	26 (13–74)	16 (10–25)		25 (21–35)
ORF3	s84	2000	1			14 (7–30)		24 (20–35)
ORF7	168	1990	12	27 (13–64)	17 (10–33)	20 (14–30)		27 (24–31)
ORF7	168	2000	58			25 (17–40)		27 (25–32)
ORF8	175	1990	21	132 (37– **∞)**	104 (19–999*)	118 (44– **∞)**		48 (37–81)
ORF8	175	2000	48			37 (22–80)		45 (35–68)

MBT: moment based temporal method; TM3: likelihood based temporal method. LDNe: the linkage disequilibrium method with bias correction; ONeSAMP is a program that uses summary statistics and approximate Bayesian computation. Lower and upper 95% confidence intervals are in brackets. *: TM3 did not produce upper confidence intervals below the imposed boundary of 999 individuals. **: no estimate possible due to too few of offspring.

Estimates from the *TM3* and *MBT* method ranged from 17 to 64 and from 27 to 45 individuals, respectively. Only estimates with informative upper boundaries for the confidence interval are included. Estimates generated by *LDNe* ranged from 14–55 individuals (excluding ORF8) and included demographic estimates of 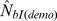 in 95%-confidence intervals in three of six cases (1 to 65) (excluding ORF8 1990 and ORF3 1990). Estimates generated by *ONeSAMP* ranged from 24–48 and were also similarly correlated to 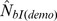 but not as well as for *LDNe*. When excluding estimates, in which upper confidence interval were uninformative, results across all the genetic estimators were fairly consistent with the exception of the MBT estimate in population ORF3.

## Discussion

The comparative approach in this study draws upon population size estimates and population genetics data of a long-lived gecko species at two points in time across four populations. Our study also incorporates genetic data on the mating system and reproductive success. As a result of this we have obtained demographic estimates of *N_bI_*. These estimates show that all populations surveyed were small with 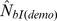 ranging from 1 to 65 individuals and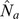, ranging from 10 to 105. The reduction in the inbreeding effective number of breeders relative to census size (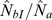) ranged from 0.1 to 0.6 with ORF7 in 2000 being the exception with a ratio of 1.1. The variance in reproductive success was the primary cause for reduction in most populations. We used four methods to estimate the genetic based *N_bI(gen)_* and *N_eV(gen)_* from the genotype data. Two of these methods require at least two samples in time, while the other two were point-in-time approaches. Genetic based estimates were fairly similar across methods and also similar to the demographic estimates (excluding those estimates, in which upper confidence interval boundaries were uninformative). For example, estimates from the point estimator ranged from 14–55 (*LDNe*) and from 24–48 individuals (*ONeSAMP*). However, temporal methods suffer from a large variation in confidence intervals and concerns about the prior information.

### Demographic-based Estimates of N_bI_

Our estimates of *N_bI_*, (1 to 65 individuals) and of *N_a_* (10 to 105) are very low for all four populations raising concerns for the long-term viability of *O. reticulata* in the Western Australian wheatbelt. The values are consistent with severe habitat fragmentation into small vegetation remnants and the observed increase in the genetic isolation of the wheatbelt populations over the last 100 years [50]. A reduction in the effective number of breeders will lead to a rapid loss of genetic variability, especially in small populations [8], and might increase the risk of extinction in both gecko species [50], [77].

### Demographic-based Estimates of N_bI_/N_a_

For most animals studied, *N_bI(demo)_* is usually lower than population size owing to unequal sex ratio and variance in reproductive success. When each demographic variable was examined independently, variance in reproductive success resulted in the largest reduction in *N_bI_/N_a_* for most populations. Unequal sex ratios had only a minor effect on the number of breeders. *N_bI_/N_a_* ranged from 0.1 - 0.6. However, one population (ORF7) was an exception, with a ratio of 1.1. The larger effective number of breeders relative to census size was probably due to its much smaller variance in reproductive success compared to the other population.

Fluctuating population size may strongly reduce *N_e_* [3], [10]. As our estimates are based on two single years only (taken ten years apart), it is possible that they may not be a good representation of the values that arise across more years, However, we do not expect this to be the case particularly because low adult mortality and high overlap of generations [46], [49] set stringent limits to annual fluctuations of population size This is reflected in similar *N_b_*/*N_a_* ratio observed for all populations across years, except for one rapidly increasing population.

These results are in line with previous studies [78], [79], [80], [81]. In particular, in Pacific salmon species with their high fecundity, variation in reproductive success can have a substantial impact on the reduction of demographic *N_e_/N_a_* [2], [10], [13], [25], [26]. The reduction observed in these studies is larger than those observed in the present study but geckos are considered to have particular low fecundity and *O. reticulata* females usually lay a clutch of two eggs per year.

The effect of the mating system (especially polygyny) on *N_e_* has been studied both theoretically [78], [82] and empirically [75], [79], [80], [83]. In feral cat populations for example, estimates of *N_e_/N_a_* were higher in a promiscuous cat population (42%) than in a polygynous one (33%) [75]. In the Italian agile frog (*Rana latastei*) *N_e_* increased with population size, but was negatively related to polygyny; as polygyny increased in large populations, this was associated with reduced *N_e_/N_a_* [80]. Compared to the species in these studies, the gecko *O. reticulata* was observed to have a particularly low fecundity and a fixed clutch size.

We also observed that variance of reproductive success in males and females was similar in all four populations of *O. reticulata*, which may be explained by the territoriality of both sexes. Higher variance of reproductive success in males has been reported regularly, for example Pacific salmon (*Salmo spec*.) and wrens (*Malurus cyaneus*) [13], [84], [85].

### Corrected Estimates of Variance in Reproductive Success

It has been suggested that uncorrected and corrected estimates of mean *k* and its variance will result in the same estimates of *N_e_* (or *N_b_*) [26], [65], [66]. This theory is based on an approximation, which can lead to substantial deviation of mean *k* and its variance in species with small population size, low reproductive output, and low variance in reproductive success. This might particularly be true for species in fragmented landscapes. Conventional uncorrected parentage assignment estimates, which are increasingly being used to measure individual reproductive success [13], [17], [24], [25], [84], are subject to errors, such as failing to identify the true parent and incorrectly assigning offspring to a parent, which can potentially bias estimates of reproductive success [27], [28]. Although methods have been developed to correct these estimates [28], they require independent information on parentage or the assumption that offspring and parents have the same probability of being sampled. Data to correct estimates is rarely available in natural populations and sampling probability is not the same for offspring and parents in this species (Henle and Gruber unpubl.) and might not be the same in many other species. Moreover, estimates of the mean reproductive success and its variance require absolute values of reproductive success, unless the probability for offspring being sampled and successfully assigned to parents is identical for all populations. This is rarely the case and similar difficulties apply for the comparison of males and females when parentage assignment success varies between sexes.

### Genetic-based Estimates of N_bI(gen)_ and N_eV(gen)_

The genetic estimates gave fairly similar results among methods apart from those estimates, whose confidence intervals were too wide to be informative. Temporal methods suffer from a large variation in confidence intervals and concerns about the prior information, which has also been reported from studies on other species [75], [86], [87], [88]. This is especially problematic when sample sizes are small, which is often the case in natural populations of endangered species. In addition, sampling should be spread out over at least four generation to reduce stochastic noise, but our data was collected only two generations apart. Another weakness of temporal genetic methods is that they ignore the effects of migration, which can bias estimates upwards. Over the long-term, constant migration can slow down the rate of change relative to what would be expected under genetic drift alone. As a consequence, the estimate of *N_eV_* will be overestimated [38], [89]. However, in previous studies we showed that *O. reticulata* exhibited extremely low levels of gene flow in fragmented habitat [50], [52] and as a result it is unlikely that *N_eV_* will be affected by immigration. In addition, the *MBT* method assumes that generations are discrete. Despite obvious limitations, the method has been applied to species with overlapping generations and the results have been considered to be satisfactory without rigorously testing for the effects of violating the discrete-generation assumption [75], [86], [89].

Regarding the point estimators, the results from *LDNe* followed the demographic estimates comparably well. *LDNe* did not produce an upper confidence limit in one of eight cases and *ONeSAMP* estimates had narrower confidence intervals. Estimates obtained with *ONeSAMP* were also not influenced by the priors. The accuracy of single-sample estimators was also compared in other studies. Using empirical data from several natterjack toad (*Bufo calamita*) populations, [44] the Bayesian method [33] and the sibship method [31] performed best (correlations with other census and effective size estimates) while the *LDNe* method gave confidence intervals for only approximately half of the populations. Nevertheless, consistent with our results, estimates for all toad populations could be obtained. That consistency was also seen in a study that estimated *N_e_* for 90 populations of four ranid frog species where values from *ONeSAMP* and the sibship method were positively correlated in all four species, had mostly similar values, and had smaller nominal confidence intervals than values from *LDNe* [90]. Several temporal and single-sample *N_e_* estimators were evaluated in natural populations of *Drosophila buzzatii. ONeSAMP* was the single-sample estimator that performed the most precise [88]. It is expected to suffer from less imprecision than the *LDNe* method, given that *ONeSAMP* is based on linkage disequilibrium plus seven other parameters that are related to *N_e_* [33], [81], [90]. However, precise does not necessarily mean best, because an estimator may be very precise but highly biased. For example, simulation studies showed that the *LDNe* method was found to have greater precision than the temporal method unless the latter was based on samples taken many generations apart [34]. These results suggest that the method might also be highly relevant for conservation studies in the future [34] An empirical study that monitors the effective population size of a brown bear (*Ursus arctos*) population found a high similarity between the estimates derived from the *ONeSAMP, LDNe* method and sibship method [91]. In addition, the study used a new Estimator by Parentage Assignment (EPA) to estimate *N_e_* and the generation interval (Wang 2010) [92]. The estimates were largely consistent with the estimates obtained from the other methods, but the comparison was complex because EPA applies to a different time period and the generation overlap had to be taken into account. However, it is the only currently method that can directly estimate *N_e_* from a single sample of genotypes in species with overlapping generations and has a considerable potential to be implemented in future monitoring [91].

Monitoring of change using a snap-shot approach would be attractive to both researchers and managers alike, but only if estimators of *N_e_* are accurate. *LDNe* and *ONeSAMP* estimates are related directly to *N_b_* [44] only if a single cohort has been sampled. Its relationship to *N_e_* is less clear should more than a single cohort of adults be sampled, as in this study [43]. Temporal estimators are closer related to *N_eV_*. However, in increasing populations *N_bI_* will be less than *N_eV_* [45], which might explain why estimates for *N_eV_* were slightly higher than for *N_bI_* in our study. The methods used in this study differ considerably in their assumptions and in the way they estimate *N_e_*. Thus the fact that we obtained fairly similar results across methods demonstrates that we can be fairly confident about the results. In addition the similarity of the point and temporal estimates suggests that the point estimates might be relatively close to *N_eV_*.

### Comparison of Demographic and Genetic-based Estimates

The genetic based estimates, in particular the single sample estimates from *ONeSAMP* and *LDNe* were in the same order of magnitude compared to the demographically estimated inbreeding effective number of breeders when excluding those estimates, in which upper confidence interval boundaries were uninformative. Three of the demographically obtained estimates fell within the 95%-confidence interval of the *LDNe* method but only one within the *ONeSAMP* method. This might be a consequence of the smaller confidence intervals of the *ONeSAMP* method and because this estimate is based on summary statistics that are influenced by both *N_eI_* and *N_eV_*. Although genetic and demographic estimates of effective population size are rare, the few that have been conducted have noted that genetically-based estimates are often substantially smaller than demographically-based estimates [14], [86], [93]. An explanation for this could be that the genetic based estimates represent *N_b_* estimates in which overlapping generations and generation length are not fully considered. Future studies are needed to better understand the reasons for these differences.

### Conclusion

In summary, the single sample estimators - *ONeSAMP* and *LDNe* - performed best among the genetic estimators of effective population size evaluated in this study, when considering confidence intervals and plausibility in relation to known population sizes. If budgets and timeframes are constrained and insufficient demographic data are available for the robust estimation of population size in endangered species, then single-sample genetic estimators may provide an acceptable alternative to monitor the change of a population. However, information on individual life history and population dynamics, such as sex ratio, variance in reproductive success, fluctuating population size, and generation length will always contribute a much more complete picture of the viability of a population. In addition to the estimate of the demographic based inbreeding effective number of breeders, robust demographic data will enable predictions about the factors that contribute to a reduction in *N_e_/N_a_*. It is encouraging that in general all estimates were reasonable and within the same order of magnitude for different methods although we are studying very small populations and the methods differ considerably in their assumptions and in the way they estimate *N_e_*. Methods to estimate *N_e_* for species with over-lapping generations have improved [18], [19], [92]. We suggest that managers should monitor both demography and genetics whenever feasible if they are to accurately assess and monitor populations of conservation concern.
